# Case Report: Vision-threatening manifestations of choroidal osteomas and papilloedema in presumed Camurati-Engelmann disease

**DOI:** 10.3389/fneur.2026.1831878

**Published:** 2026-07-28

**Authors:** Anika Rao, Irina Cristescu, Hibba Quhill, Umiya Harley, Satheesh Ramalingam, Georgios Tsermoulas, Susan P. Mollan, Gabriele Berman

**Affiliations:** 1 Department of Clinical Education, Leicester Royal Infirmary, University Hospitals of Leicester NHS Trust, Leicester, United Kingdom; 2 Ocular Oncology Service, Department of Ophthalmology, Royal Hallamshire Hospital, Sheffield, United Kingdom; 3 Department of Neuroradiology, University Hospitals Birmingham NHS Foundation Trust, Birmingham, United Kingdom; 4 Department of Neurosurgery, University Hospitals Birmingham NHS Foundation Trust, Birmingham, United Kingdom; 5 Metabolism and Systems Science, School of Medical Sciences, University of Birmingham, Birmingham, United Kingdom; 6 Department of Ophthalmology, Queen’s University, Kingston, ON, Canada; 7 Birmingham Neuro-Ophthalmology, University Hospitals Birmingham NHS Foundation Trust, Birmingham, United Kingdom

**Keywords:** anti-vegf, Camurati-Engelmann disease, choroidal osteoma, papilloedema, photodynamic therapy

## Abstract

Camurati–Engelmann Disease (CED) is a rare sclerosing bone dysplasia in which skull-base hyperostosis may result in neuro-ophthalmic complications. We report a 21-year-old woman with presumed CED and known bilateral choroidal osteomas who presented with bilateral optic nerve swelling. Neuroimaging demonstrated diffuse cranial hyperostosis with optic canal narrowing but no definitive optic nerve compression or venous sinus thrombosis. Lumbar puncture confirmed raised intracranial pressure with an opening pressure of 33 cm H₂O. Optical coherence tomography revealed right eye subretinal fluid without choroidal neovascular membrane. Papilloedema improved initially with acetazolamide, but treatment was changed to oral furosemide due to intolerance to acetazolamide. There was continued visual decline in association with progressive subretinal fluid. Right eye intravitreal bevacizumab was ineffective, while fovea-sparing photodynamic therapy achieved resolution of subretinal fluid but was complicated by retinal pigment epithelial and photoreceptor loss. At 2.5-year follow-up, visual acuity was reduced in both eyes, with mild bilateral chronic papilloedema and enlargement of choroidal osteomas. This case illustrates multifactorial visual loss in CED with skull base hyperostosis arising from raised intracranial pressure and co-existing retinochoroidal pathology. This is the first documented case of CED with bilateral choroidal osteomas and highlights the role of multimodal imaging and multidisciplinary collaboration.

## 1 Introduction

Camurati-Engelmann Disease (CED) also known as progressive diaphyseal dysplasia is an autosomal dominant condition caused by gene variants in transforming growth factor beta 1 (*TGFB*1) gene (1). Camurati-Engelmann Disease is rare with just over 300 reported cases (2) and an estimated occurrence of approximately 1 per 1,000,000 (3). It typically presents in childhood with limb pain, waddling gait and muscle weakness. It is characterised by hyperostosis affecting the long bones and the skull leading to macrocephaly and frontal bossing (1, 2). The ophthalmic manifestations of CED include exophthalmos, papilloedema and optic nerve atrophy and are thought to be attributable to cranial hyperostosis, increased intracranial pressure and optic canal stenosis with or without compressive optic neuropathy (2).

Choroidal osteoma is a benign ossifying tumour characterised by mature bone replacing the choroid (4, 5). Visual decline can result from lesion enlargement, decalcification, retinal pigment epithelium (RPE) atrophy or subretinal fluid (SRF) with or without a choroidal neovascular membrane (CNVM) (6, 7). Secondary CNVM have been reported in 31–48% of eyes (5, 8). Subretinal fluid in absence of a CNVM has been reported in 15% of cases occurring most commonly in osteomas without decalcification or RPE atrophy and less commonly in cases with RPE atrophy with and without decalcification (8). Increased choroidal osteoma thickness with displacement of choriocapillaris was seen in eyes with SRF without CNVM (8). There is no consensus on the best management for choroidal osteomas; however, treatment options currently include intravitreal anti-vascular endothelial growth factor (VEGF) and photodynamic therapy (PDT).

We present a unique case of a young woman with presumed CED, papilloedema and bilateral choroidal osteomas who developed progressive vision loss. This is the first documented case of CED with co-existing choroidal osteomas highlighting management challenges and need for a multidisciplinary team collaboration.

## 2 Case report

### 2.1 Neuro-ophthalmology

**Figure 1:**
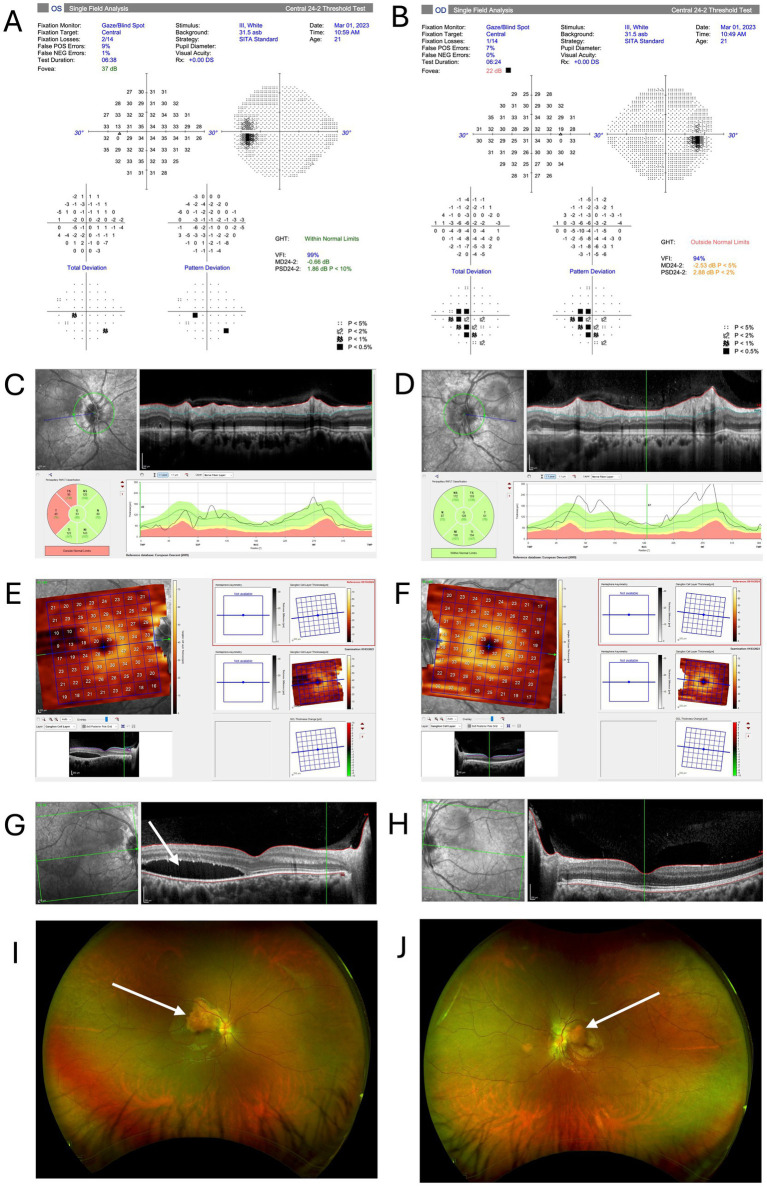
**(A)** 24–2 Humphrey visual fields at presentation showing left eye within normal limits. **(B)** 24–2 Humphrey visual fields at presentation showing right paracentral scotoma on the pattern deviation plot. **(C)** OCT retinal nerve fibre layer scan at presentation showing near normal retinal nerve fibre layer thickness right eye. **(D)** OCT retinal nerve fibre layer scan at presentation showing mildly increased retinal nerve fibre layer thickness left eye. **(E)** OCT posterior pole scan at presentation showing mild ganglion cell layer loss right eye. **(F)** OCT posterior pole scan at presentation showing preserved ganglion cell layer thickness left eye. **(G)** OCT posterior pole macula scan at presentation showing subretinal fluid in the right eye (white arrow). **(H)** Normal OCT posterior pole macula scan at presentation left eye. **(I)** Ultra-widefield fundus imaging at presentation showing right choroidal osteoma (white arrow). **(J)** Ultra-widefield fundus imaging at presentation showing left choroidal osteoma (white arrow).

A 21-year-old woman was referred to neuro-ophthalmology clinic with bilateral optic nerve swelling. Her past medical history was notable for clinically diagnosed CED in childhood following lower limb pain, waddling gait and characteristic radiographic findings. She had a past medical history of atrial tachycardia, chronic migraine, and delayed puberty which required pubertal induction at age 18. At presentation she described a six-month history of daily headaches with exacerbations accompanied by photophobia and phonophobia. She reported pulse-synchronous tinnitus and transient visual obscurations precipitated by straining. Her blood pressure was 164/89 mmHg, pulse 79 beats/min, height 156 cm, weight 39 kg (BMI 16 kg/m^2^). Visual acuity was 6/6 in both eyes with preserved colour vision and no relative afferent pupillary defect. Humphrey visual fields (HVFs) demonstrated a right paracentral scotoma (Figures 1A,B). Examination revealed mild bilateral optic nerve swelling. Optical coherence tomography (OCT) (Spectralis OCT, Heidelberg Engineering, Germany) showed corresponding retinal nerve fibre layer (RNFL) thickening (Figures 1C,D) and very small volume peripapillary hyperreflective ovoid mass-like structures, and mild ganglion cell layer (GCL) thinning right eye with preserved GCL left eye (Figures 1E,F). OCT macula showed SRF right eye with no signs of choroidal neovascular membrane (CNVM) (Figures 1G,H). Optos ultra-widefield retinal imaging (Optos Inc., United Kingdom) revealed bilateral posterior pole yellow white lesions in keeping with choroidal osteomas (Figures 1I,J).

### 2.2 Neuroradiology

**Figure 2:**
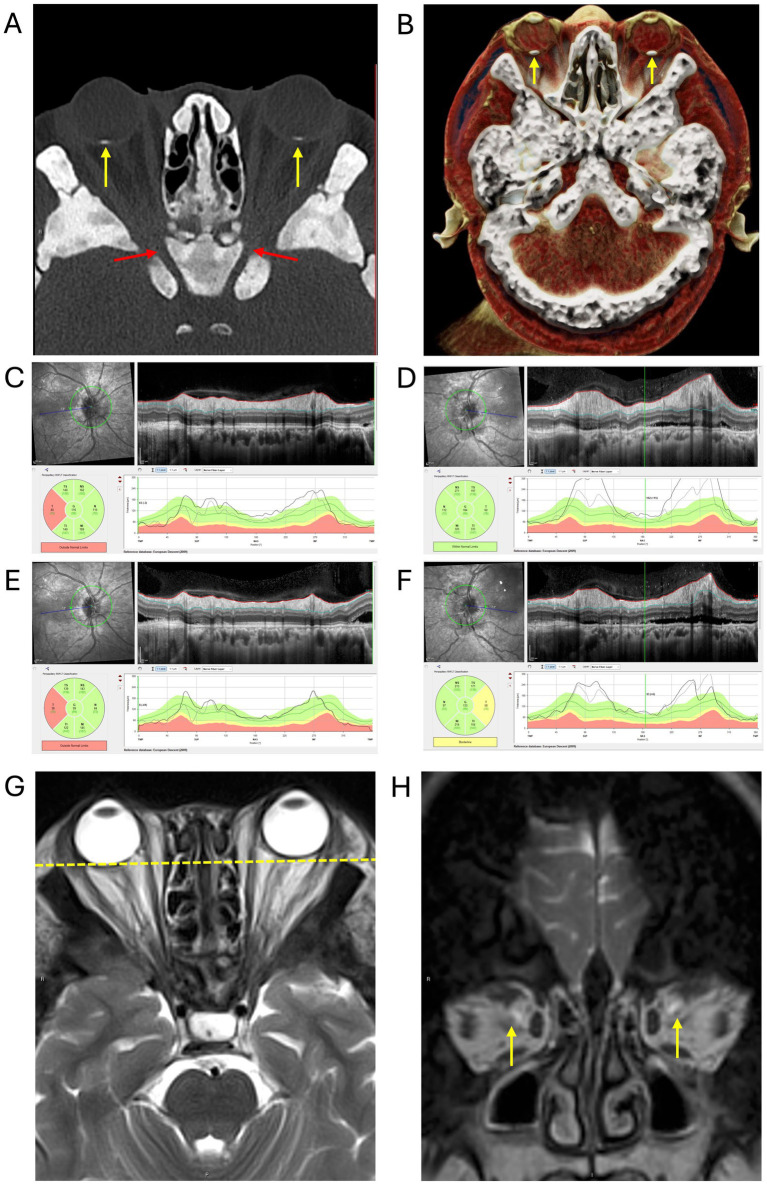
**(A)** CT head showing bilateral optic canal narrowing (red arrows) and bilateral choroidal osteomas (yellow arrows). **(B)** CT volume rendering technique showing extensive cranial hyperostosis and bilateral choroidal osteomas (yellow arrows). **(C)** OCT retinal nerve fibre layer scan showing increase in retinal nerve fibre layer thickness right eye. **(D)** OCT retinal nerve fibre layer scan showing increase in retinal nerve fibre layer thickness left eye and small amount of peripapillary subretinal fluid. **(E)** Reduction in right eye retinal nerve fibre layer thickness following acetazolamide 250 mg twice daily. **(F)** Reduction in left eye retinal nerve fibre layer thickness following acetazolamide 250 mg twice daily. Of note there is peripapillary subretinal fluid present (most likely due to macular subretinal fluid), which would impact the total retinal thickness measurement. However, the retinal nerve fibre layer thickness used as measure for papilloedema in this case was not influenced by peripapillary fluid as evidenced by satisfactory RNFL segmentation. RNFL segmentation was checked for all RNFL scans and if segmentation error present, manual resegmentation was performed. **(G)** Axial MRI T2 weighted imaging showing bilateral proptosis (yellow dashed line). **(H)** Coronal MRI T2 weighted imaging showing bilateral optic nerve atrophy (yellow arrows).

Previous investigations included CT head demonstrating extensive cranial hyperostosis, narrowing of optic canals and posterior globe calcifications. (Figures 2A,B). Venous imaging showed no venous sinus thrombosis.

### 2.3 Neuro-ophthalmology

Lumbar puncture opening pressure was 33 cmH₂O, cerebrospinal fluid composition was normal and treatment with acetazolamide 250 mg twice daily increasing to 500 mg twice daily was started. The patient discontinued acetazolamide after several weeks of treatment due to intolerance from palpitation symptoms.

### 2.4 Neuro-ophthalmology

Six months later she re-presented with worsening vision, headaches, and pulse-synchronous tinnitus. Visual acuity was 6/12 bilaterally, HVFs showed worsening of the right scotoma and OCT imaging confirmed increased papilloedema (Figures 2C,D). Acetazolamide 250 mg twice daily was re-trialled, resulting in improvement in papilloedema (Figures 2E,F), but was suspended after one month in accordance with the patient’s wishes. Blood tests showed raised serum alkaline phosphatase (608 IU/L) consistent with CED, normal adjusted calcium concentration (2.25 mmol/L) and mild microcytic anaemia with normal haemoglobin (121 g/L). Topiramate treatment was discussed with patient, however given UK regulatory advice with safety measures for topiramate (mandating Pregnancy Prevention Programme) and her significant concerns over side effects, conditions were not met for prescription and topiramate was not prescribed as per the patient wishes.

### 2.5 Neuroradiology

Repeat CT head imaging showed extensive hyperostosis and skull base expansion with narrowing of optic canals but no definitive radiological mechanical compression of the optic foramina. Magnetic Resonance Venography (MRV) showed patent dural venous sinus. MRI head showed proptosis and bilateral optic nerve atrophy (Figures 2G,H).

### 2.6 Neurosurgery

Neurosurgery were consulted for possibility of implantation of intracranial pressure monitor, however, this and any surgical procedure requiring drilling of skull would have been technically challenging due to degree of hyperostosis and was deemed not in patient’s best interest as there was previous documentation of raised lumbar puncture opening pressure.

### 2.7 Medical retina

**Figure 3:**
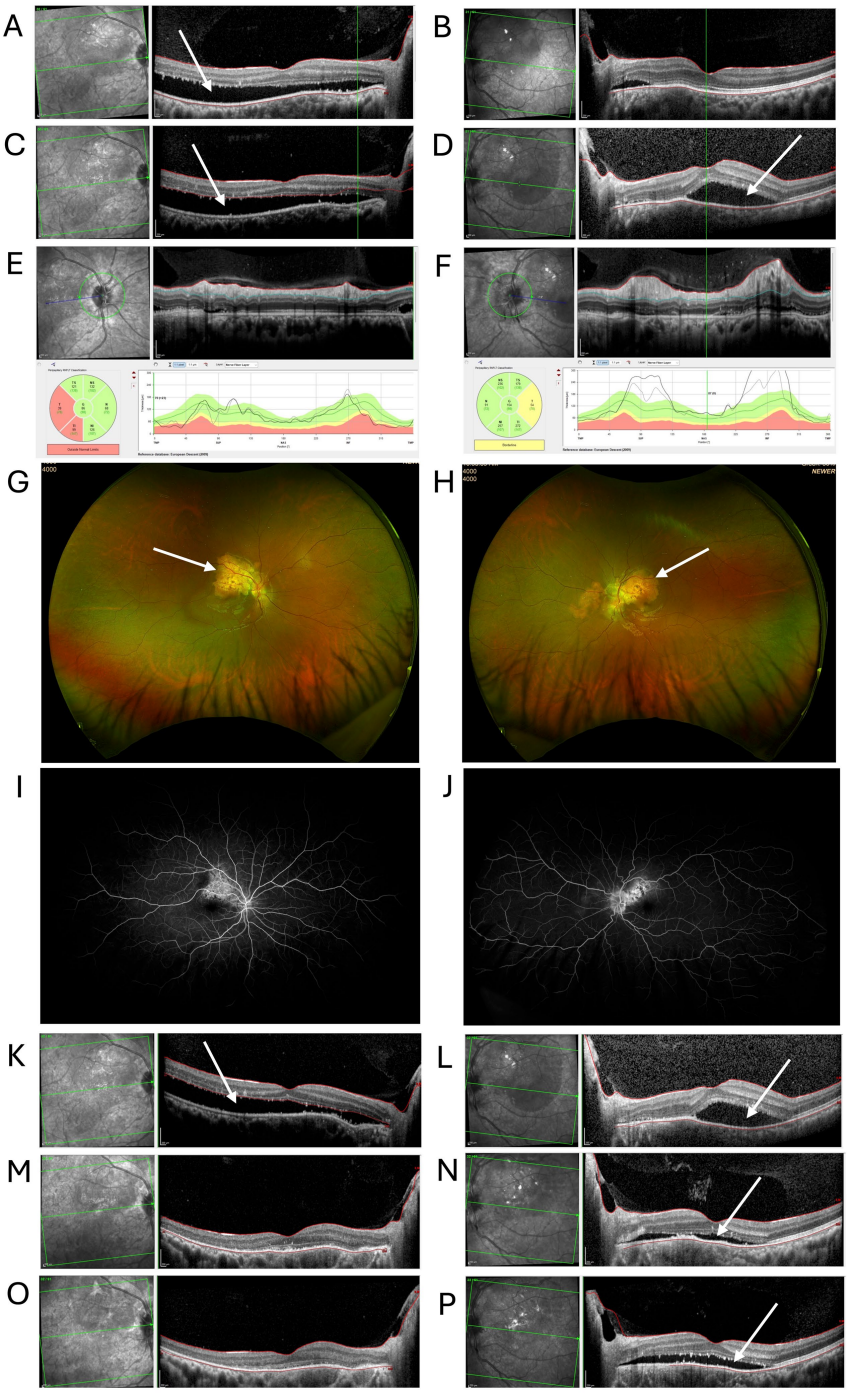
**(A)** Posterior pole OCT macula right eye showing increasing subretinal fluid (white arrow) extending sub-foveally. **(B)** Posterior pole OCT macula left eye showing mild subretinal fluid peripapillary. **(C)** Posterior pole OCT macula right eye showing subretinal fluid (white arrow) following three intravitreal injections of bevacizumab. **(D)** Posterior pole OCT macula left eye showing increasing subretinal fluid (white arrow) following three intravitreal injections of bevacizumab to right eye. **(E)** OCT retinal nerve fibre layer scan right eye showing stable retinal nerve fibre layer thickness following three intravitreal injections of bevacizumab. **(F)** OCT retinal nerve fibre layer scan left eye (untreated) showing increased retinal nerve fibre layer thickness following three intravitreal injections of bevacizumab to right eye. **(G)** Ultra-widefield fundus imaging showing increase in size of right choroidal osteoma (white arrow). **(H)** Ultra-widefield fundus imaging showing increase in size of left choroidal osteoma (white arrow). **(I)** Fundus fluorescein angiography right eye demonstrating no choroidal neovascular membrane. **(J)** Fundus fluorescein angiography left eye demonstrating no choroidal neovascular membrane. **(K)** Posterior pole OCT macula showing right eye sub-retinal fluid (white arrow) pre-photodynamic therapy. **(L)** Posterior pole OCT macula showing left eye sub-retinal fluid (white arrow) at time of right eye photodynamic therapy. **(M)** Posterior pole OCT macula right eye 3 months post-photodynamic therapy showing resolution of sub-retinal fluid. **(N)** Posterior pole OCT macula left eye (untreated) showing sub-retinal fluid (white arrow) at 3 months post-photodynamic therapy to right eye. **(O)** Posterior pole OCT macula right eye 12 months post-photodynamic therapy showing absence of sub-retinal fluid. **(P)** Posterior pole OCT macula left eye (untreated) showing sub-retinal fluid (white arrow) at 12 months post-photodynamic therapy to right eye.

Three months later patient reported subjective reduction in right eye vision. Visual acuity was 6/9 in both eyes, optic disc swelling had improved bilaterally, but OCT revealed SRF extending sub-foveally in the right eye (Figures 3A,B). OCT angiography excluded CNVM, and after risk benefit discussion empiric treatment with monthly intravitreal bevacizumab injection right eye was started.

### 2.8 Neuro-ophthalmology

Oral furosemide 20 mg daily was started with subsequent reduction in optic nerve swelling. After completion of monthly intravitreal bevacizumab for three months there was continued vision decline (6/30 RE, 6/18 LE), OCT showed increased SRF right eye and new SRF left eye (Figures 3C,D) and unchanged RNFL thickness (Figures 3E,F). The choroidal osteomas had increased in size over time (Figures 3G,H). The patient was referred to ocular oncology.

### 2.9 Ocular oncology

At time of referral, visual acuity was 6/24 and 6/18. Fundus fluorescein angiography showed patchy hyperfluorescent choroidal filling pattern with late diffuse staining without CVNM (Figures 3I,J). After a risk benefit discussion, the patient elected to proceed with a single session fovea sparing PDT (83 s at 50 mJ/cm) targeting right choroidal osteoma and for observation of the left eye. Posterior pole OCT macula showed bilateral sub-retinal fluid at the time of right-eye photodynamic therapy, right eye more than left eye (Figures 3K,L). Three months after PDT there was reduction in SRF right eye (Figures 3M,N) and complete resolution of SRF right eye at six months. One year following right eye PDT, visual acuity was 6/24 both eyes with resolution of SRF and outer retinal changes right eye (Figures 3O,P).

### 2.10 Neuro-ophthalmology

**Figure 4:**
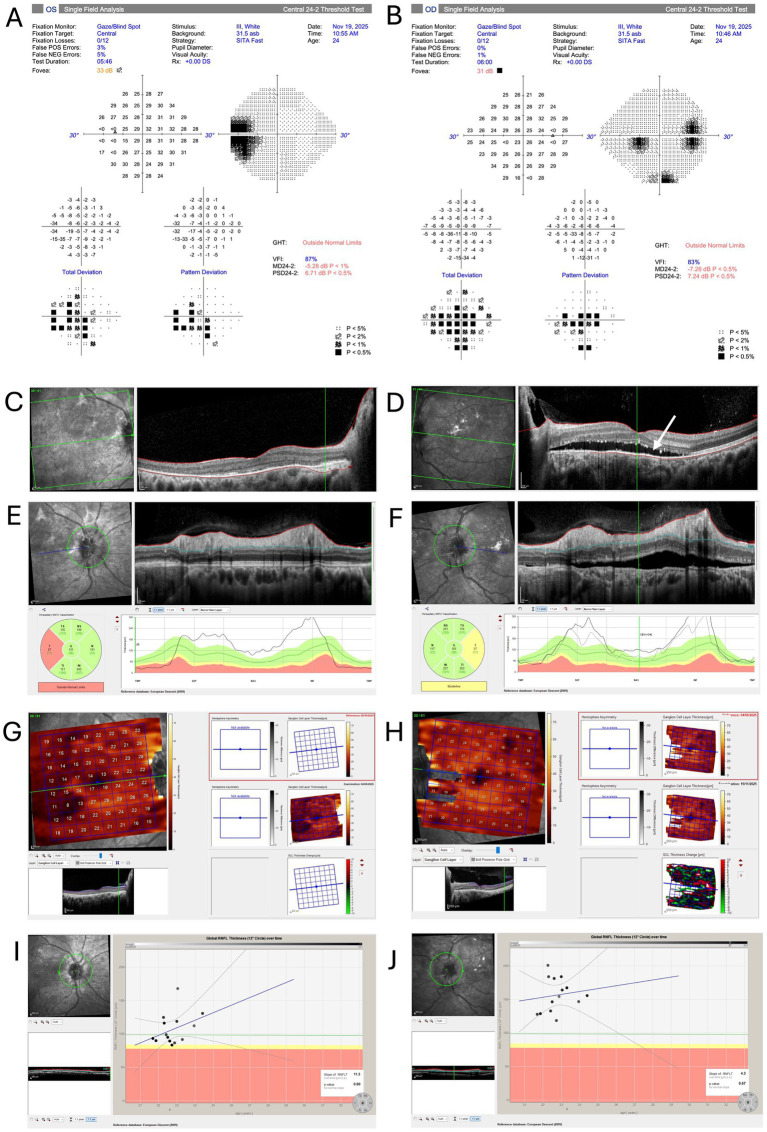
**(A)** 24–2 Humphrey visual fields left eye at 2.5 years following presentation showing an enlarged blind spot on pattern deviation plot. **(B)** 24–2 Humphrey visual fields right eye at 2.5 years following presentation showing right paracentral scotoma on pattern deviation plot. **(C)** Posterior pole OCT macula showing resolution of subretinal fluid and presence of outer retinal atrophy in right eye at 2.5 years following presentation. **(D)** Posterior pole OCT macula showing persistent subretinal fluid (white arrow) in left eye at 2.5 years following presentation. **(E)** OCT retinal nerve fibre layer scan right eye showing persistent mild optic nerve swelling at 2.5 years following presentation. **(F)** OCT retinal nerve fibre layer scan left eye showing persistent mild optic nerve swelling and peripapillary subretinal fluid at 2.5 years following presentation. **(G)** Posterior pole OCT macula showing ganglion cell layer thinning right eye at 2.5 years following presentation. **(H)** Posterior pole OCT macula showing ganglion cell layer thinning left eye at 2.5 years following presentation. **(I)** Global retinal nerve fibre layer thickness plot showing fluctuations in right eye global retinal nerve fibre layer thickness over 2.5 years. **(J)** Global retinal nerve fibre layer thickness plot showing fluctuations in left eye global retinal nerve fibre layer thickness over 2.5 years.

At 2.5 years after initial presentation her examination recorded a visual acuity of 6/24 with dyschromatopsia and mild chronic bilateral optic nerve swelling. Visual fields showed a paracentral scotoma right eye and enlarged blind spot left eye (Figures 4A,B). OCT macula showed resolution of SRF right eye with RPE atrophy and subfoveal SRF left eye (Figures 4C,D). OCT RNFL scans showed stable mild bilateral optic nerve swelling (Figures 4E,F) and GCL thinning in both eyes (Figures 4G,H). She has remained on oral furosemide 20 mg daily with fluctuating RNFL thickness (Figures 4I,J).

## 3 Discussion

Cranial involvement in CED can lead to vision-threatening complications. Skull base hyperostosis has been reported in over half of CED cases, with papilloedema and visual changes occurring in approximately 5% (2). It is thought that ophthalmic symptoms may arise from compressive optic neuropathy secondary to optic foramen narrowing or papilloedema due to raised intracranial pressure (ICP) secondary to diffuse hyperostosis reducing cranial vault volume, jugular foramen stenosis or venous outflow obstruction (2, 9). In our case there was progressive visual loss with clinical features of raised ICP confirmed by LP, in the absence of venous outflow obstruction or definite mechanical compression of the optic canal (however evidence of optic canal stenosis). We consider that the mechanism of raised ICP is likely due to diffuse cranial hyperostosis causing a reduction in intracranial volume (9, 10).

There is paucity of evidence on medical management of papilloedema in CED, and acetazolamide has been reported to resolve papilloedema (9, 10). In our case, furosemide proved an alternative in managing papilloedema, as the patient was intolerant of acetazolamide. The use of oral glucocorticoids likely remains controversial where the evidence details one patient with resolved papilloedema (11) and a larger series suggesting no improvement in outcomes in symptomatic skull base disease (2).

Surgical decompression has been reported as an option in carefully selected patients with progressive skull base involvement (2). However, these procedures carry high risk of irreversible iatrogenic vision loss and are technically demanding due to extensive hyperostosis with considerable risk of recurrence from bony regrowth (1, 2).

Visual loss in this patient was likely multi-factorial due to retinal/choroidal and optic nerve involvement. Chronic papilloedema causing optic nerve atrophy has been reported in previous cases with CED (1, 2) and was evidenced in this case by ganglion cell depletion and an element of compressive optic neuropathy in context of optic canal stenosis cannot be excluded. Additionally, there was growth of choroidal osteomas and subfoveal SRF in absence of CNVM. In pre-OCT era a cohort of patients with choroidal osteomas was observed for a median of 10 years and documented osteoma growth in 41% via colour fundus photography (12). Shields *et al.* reported osteoma growth in 51% of eyes at 10 years and CNVM in 31% of eyes (5). There is no consensus on the best treatment of choroidal osteomas, and treatment strategies are aimed at CNVM and/or SRF. The use of anti-VEGF for SRF without CNVM in choroidal osteoma remains unpredictable and there were no significant visual acuity gains in our patient, although we note no worsening. Reports have shown partial or complete resolution of SRF in absence of CNVM in patients with choroidal osteomas (13) however overall, evidence suggests best response if CNVM is present (7, 14, 15). PDT has also been used for SRF without CNVM (16) and SRF with CNVM (17, 18). However, the treatment of subfoveal CNVM is complex and may worsen visual acuity due to decalcification and associated RPE loss (5, 7). Factors including presence/absence of CNVM, location of CNVM and risk of vision loss must be taken into consideration when discussion management options and complexities with patients presenting with SRF in presence of choroidal osteoma. There was resolution of SRF following PDT in our case but also development of outer retinal changes including RPE loss. The uncertain visual outcomes and possible complications of PDT further emphasise the importance of patient counselling.

## 4 Conclusion

This case illustrates how cranial hyperostosis and coexisting choroidal osteomas can together drive progressive visual decline in CED through several pathways of optic neuropathy and retinochoroidal pathology. Medical management with acetazolamide or alternative diuretics can be used to manage papilloedema. The co-occurrence of choroidal osteomas with SRF without CNVM presented further challenges and were not responsive to intravitreal bevacizumab. There was resolution of SRF following PDT but subsequent decline of vision in setting of RPE loss and optic neuropathy. Multimodal imaging and multi-disciplinary approach were essential in this case to guide individualised management.

## Data Availability

The original contributions presented in the study are included in the article/supplementary material, further inquiries can be directed to the corresponding author/s.
